# Investigating cross‐contamination by yeast strains from dental solid waste to waste‐handling workers by DNA sequencing

**DOI:** 10.1002/mbo3.554

**Published:** 2017-12-26

**Authors:** Cristina Dutra Vieira, Thaysa Leite Tagliaferri, Maria Auxiliadora Roque de Carvalho, Maria Aparecida de Resende‐Stoianoff, Rodrigo Assuncao Holanda, Thais Furtado Ferreira de Magalhães, Paula Prazeres Magalhães, Simone Gonçalves dos Santos, Luiz de Macêdo Farias

**Affiliations:** ^1^ Laboratory of Oral Microbiology and Anaerobes Department of Microbiology Institute of Biological Sciences Federal University of Minas Gerais ‐ Avenida Antônio Carlos Belo Horizonte Brazil; ^2^ Mycology Laboratory Department of Microbiology Institute of Biological Sciences Federal University of Minas Gerais ‐ Avenida Antônio Carlos Belo Horizonte Brazil; ^3^ Parasite Biology Laboratory CEUMA University ‐ Rua dos Castanheiros São Luís Brazil

**Keywords:** antifungal resistance, environmental microbiology, fungal genes, health care solid waste, yeast

## Abstract

Trying to widen the discussion on the risks associated with dental waste, this study proposed to investigate and genetically compare yeast isolates recovered from dental solid waste and waste workers. Three samples were collected from workers' hands, nasal mucosa, and professional clothing (days 0, 30, and 180), and two from dental waste (days 0 and 180). Slide culture, microscopy, antifungal drug susceptibility, intersimple sequence repeat analysis, and amplification and sequencing of internal transcribed spacer regions were performed. Yeast strains were recovered from all waste workers' sites, including professional clothes, and from waste. Antifungal susceptibility testing demonstrated that some yeast recovered from employees and waste exhibited nonsusceptible profiles. The dendrogram demonstrated the presence of three major clusters based on similarity matrix and UPGMA grouping method. Two branches displayed 100% similarity: three strains of *Candida guilliermondii* isolated from different employees, working in opposite work shifts, and from diverse sites grouped in one part of branch 1 and cluster 3 that included two samples of *Candida albicans* recovered from waste and the hand of one waste worker. The results suggested the possibility of cross‐contamination from dental waste to waste workers and reinforce the need of training programs focused on better waste management routines.

## 1 INTRODUCTION

Waste is considered by Muhamedagic, Muhamedagic, and Masic (2009) a direct consequence of human activity. The authors reported that dental staff generates large amounts of waste, which may be contaminated with body fluids. According to World Health Organization (WHO, 2014), hazardous health care wastes are those that contain pathogens (bacteria, viruses, parasites, or fungi) in sufficient concentration or quantity to cause disease in susceptible hosts. Health care waste is generated during the diagnosis, treatment, and immunization of human beings or animals (Nabizadeh, Faraji, & Mohammadi, 2014) and comes from many sources, including dental clinics (WHO, 2014). In Brazil, dental waste remains regulated by the same Federal Resolutions, which control medical waste (National Health Surveillance Agency ‐ Anvisa, 2004; National Concil of Environment ‐ Conama, 2005). Caniato, Tudor, and Vaccari (2015) emphasized that a serious lack of reliable data regarding waste generation and its characteristics is a global issue, making the appropriate management solutions very difficult. This information is in agreement with Muhamedagic et al. (2009) who pointed out that the correct management and safe disposal of dental waste is one of the key ecological challenges of the modern world. There are some studies focusing on hospital and dental waste, although very few discuss specific aspects such as their potential biological risks, specially their fungi content. Previous results of our group (Vieira et al., 2010, 2011) and other studies (Madsen, Alwan, Ørberg, Uhrbrand, & Jørgensen, 2016) proved the presence of different species of viable bacteria and also of some yeast inside biomedical waste. To widen the discussion on the possible risks to waste workers, this study proposed to investigate fungal strains recovered from dental waste, comparing with those samples isolated from waste workers' tissues (hands and nasal mucosa) and their work wear. The susceptible profile of fungi samples to recommended antimicrobials was also surveyed.

## 2 EXPERIMENTAL PROCEDURES

### 2.1 Institutional characteristics and sampling

The study was performed in a Public Dental Healthcare Service in Belo Horizonte, state of Minas Gerais, Brazil. The team responsible for processing environmental surfaces and waste collection was composed of 12 members, 6 in the first work shift (from 06:00 a.m. to 03:00 p.m.) and 6 in the second shift (01:00 p.m. to 10:00 p.m.). To investigate the microbial load of waste workers, three complete samplings were performed on days 0, 30, and 180. Hands and coats were sampled using the methodology proposed by Snyder et al. (2008). Hand testing was carried out by moving three times a saline‐moistened sterile swab across the dorsum of each finger and finished by drawing two circles on the palm, covering as much of the palm as possible. Sample collection begun with the nondominant hand and finished with the dominant one. Swabs were taken from professional coats by drawing a “W” with the saline‐moistened swab on the belt line area. Two samples were collected from nasal mucosa according to Neves (2007). The swab was inserted into the anterior nare (<1 cm) and rotated gently for 10 sec. All swabs were inserted into a transport tube, properly labeled, and immediately transported to the laboratory. Sampling was performed by a trained professional. Waste sampling took place on days 0 and 180 adopting previously published methodology (Vieira et al., 2011). In this study, we considered the three main categories proposed by Kizlary, Iosifidis, Voudrias, and Panagiotakopoulos (2005) to separate dental solid waste: infectious waste, noninfectious waste, and domestic‐type waste. To properly investigate the infectious category, dental solid waste produced in 24 hr was transferred to the waste storage room and were visually inspected and manually separated by specially trained personnel, using personal protective equipments (PPE). PPE included gloves, masks, goggles, gowns, and protective clothing. This waste category included those materials that were suspected to contain pathogens and that poses a risk of disease transmission (WHO, 2014). These materials represented 19.6 kg (mean values for the two samples) and represent 67.3% of the total amount of waste generated in 24 hr. From this portion, randomly 500 mg sample was obtained and placed in a sterilized container; to enhance microbial recovery, 500 ml of sterile saline solution supplemented with l‐cysteine hydrochloride monohydrate (InLab^®^) and Tween 80 with pH adjusted to 7.0–7.2 was added (Vieira et al., 2011). Waste sample was aseptically mixed every 15 min and after 1 hr contact, 150 ml of the leached liquid was collected inside a sterile bottle. The flask was immediately transported to the laboratory and one aliquot of 0.1 ml was streaked onto Sabouraud dextrose agar (SDA, Difco^™^). Morphologically different colonies were isolated as pure cultures for later investigation (Vieira et al., 2010). All yeast isolates except two were recovered from SDA. Yeast identification was obtained using conventional, automated, and genetic techniques.

### 2.2 Routines of waste management

Dental waste management was implemented in 2001 and approved by the local government in the same year, in 2008 and in 2015. Wide wheeled containers (capacity 500 and 1,000 L), small washable and colored containers with pedals (100 L) and gram precision digital scale (capacity: up to 60 kg) were acquired. All infectious waste coming from dental offices was discarded inside small orange containers lined with white plastic bags with the international symbol for biological hazard. At the end of the work shift (11:30 a.m., 16:30 p.m., and 08:30 p.m.) the bags were collected and transported to the waste storage room. These plastic bags were daily weighed and sent for treatment by incineration. Common waste was discarded inside blue plastic bags disposed of inside small white containers and was collect by the government. All small containers were externally labeled as “infectious” or “common” waste depending on its content. All employees received training on segregation, collection, storage, transportation, and waste disposal, and a permanent educational program has been established with discussion, lectures, and seminars. All 12 waste workers were involved in waste collection from dental offices and also transport three times a day the containers to the waste storage room. All daily routines had been performed using protective clothing, gloves, masks, goggles, and gowns.

### 2.3 Conventional and automated identification

Slide culture and microscopy were performed according to Sousa et al. (2016). One fragment of corn meal agar added with Tween 80 was placed over a sterile glass slide. After inoculating yeast isolates by making three central grooves, a sterile cover slip was placed on the agar surface. The culture was incubated inside a sterile Petri dish with a piece of cotton saturated with sterile water to maintain humidity at 28°C for nearly 7 days. From the fifth day of incubation, yeast growth was observed to perform the morphological identification. Yeast were speciated by visualizing the formation of pseudohyphae, hyphae, chlamydospores, and also the precise blastoconidium distribution over this structures. All samples isolated were identified by the automated system Vitek^®^ (bioMérieux) using Vitek^®^2 YST ID cards (Graf, Adam, Zill, & Go, 2000). Scores above 80% were considered for acceptable identification. *Candida parapsilosis* ATCC 22019 was included as control.

### 2.4 Antifungal drug susceptibility

Antifungal susceptibility testing was performed according to the Clinical and Laboratory Standards Institute (CLSI, 2008) updated to CLSI 2012 parameters reported by some published papers (Alastruey‐Izquierdo et al., 2015; Almeida et al., 2012; Nunes et al., 2013; Orasch et al., 2014; Ramos et al., 2015; Santos et al., 2014). The last edition of CLSI revised the azole and echinocandin clinical breakpoints against *Candida* species. The new breakpoints are now both drug and species specific, whereas the previous breakpoints were not (Fothergill, Sutton, McCarthy, & Wiederhold, 2014). Five antifungal drugs were selected considering the literature and clinical recommendations. Final drug concentrations were of 0.125–64 μg/ml for fluconazole and 5‐fluorocytosine, 0.015–8.0 μg/ml for caspofungin, and 0.0313–16 μg/ml for voriconazole and amphotericin B. When the MIC was lower than the breakpoint defined by CLSI, the isolate was considered susceptible, and if the MIC was higher than the breakpoint, the isolate was considered nonsusceptible. Samples considered dose‐dependent susceptible, intermediate, and resistant were included in the last group (Orasch et al., 2014; Santos et al., 2014). *Candida parapsilosis* ATCC 22019 was included in all experiments as the quality control strain.

### 2.5 Genetic investigation

#### 2.5.1 DNA isolation

Initially, yeast cells were disrupted by heating and freezing steps, under denaturing conditions. Briefly, after growing on SDA at 28°C for 48 hr, 50–100 mg of biomass of each sample were suspended in 500 μl of extraction buffer (250 mmol/L Tris‐HCl; 25 mmol/L EDTA; 0.5% (w/v) SDS; 250 mmol/L NaCl; pH 8.0), then vortexed for 1 min, and heated at 80°C for 20 min followed by freezing at −20°C for the same period. Then, the samples were centrifuged at 1,077*g* for harvesting the supernatant. DNA isolation from the upper aqueous phase was carried out by using a chloroform–isoamyl alcohol mixture (24:1) (14,475 g; 25°C for 10 min), followed by the DNA precipitation with 0.7 vol of isopropanol (14,475 g; 4°C for 30 min), and a washing step with 70% (v/v) ethanol (14,475 g; 4°C for 10 min). At last, DNA was suspended in 40 μl of sterile ultrapure water, and after measuring its concentration by using Nanodrop 1,000 instrument (Thermo Fisher Scientific), it was stored at −20°C.

#### 2.5.2 Amplification and sequencing of ITS regions

The ITS1‐5.8S‐ITS2 rDNA locus was amplified by PCR using ITS1 (5′‐TCCGTAGGTGAACCTGCGG‐3′) and ITS4 (5′‐TCCTCCGCTTATTGATATGC‐3′) oligos, according to Brilhante et al. (2005), with modifications. The reaction was performed in a total volume of 25 μl programmed as follows: an initial denaturation for 4 min at 94°C, followed by 40 cycles (1 min at 94°C, 1 min at 55°C, and 2 min at 72°C) and a final extension of 5 min at 72°C. Then, the amplicons were submitted to electrophoresis in 1% agarose gel stained with ethidium bromide to be visualized under ultraviolet transilluminator. For sequencing of ITS1‐5.8S‐ITS2 rDNA, the amplicons were purified by precipitation with PEG (Sambrook & Russell, 2001), and DNA concentration was estimated by electrophoresis based on intensity of a DNA standard of known concentration. DNA sequencing was performed by Myleus Biotechnology (www.myleus.com) by using 3730 DNA Analyzer (Thermo Fisher Scientific), BigDye terminator v 3.1, and POP7 polymer. The quality of chromatograms was judged according to Phred software parameters (http://www.phrap.org/).

#### 2.5.3 Intersimple sequence repeat

Genetic characterization was performed by PCR using (GTG)_5_ (5′‐GTG GTG GTGGTGGTG‐3′) oligo, according to Roque, Vieira, Rato, and Luz‐Martins (2006), with modifications. Amplification reaction was performed in a total volume of 25 μL as follows: an initial denaturation for 10 min at 95°C, followed by 35 cycles (30 s at 95°C, 1 min at 39°C, and 2 min at 72°C) and a final extension for 5 min at 72°C. The DNA band patterns were visualized by electrophoresis as previously described and photodocumented. For DNA polymorphism analysis, a binary data matrix was built based on the presence and absence of DNA bands and considering their electrophoretic migration patterns. These data were submitted to NTSYS.PC (Numerical Taxonomy System, Applied Biostatistics, version 2.1) software to build a similarity matrix based on Jaccard's coefficient. Then, similarity matrix was grouped by UPGMA method (unweighted pair group method with arithmetical averages) for building dendrograms.

#### 2.5.4 Ethical aspects

Ethics approval for the study was granted by the Ethics Committees of the Military Hospital (protocol number 006/2013) and Federal University of Minas Gerais (Certificate of Presentation for Ethics Appreciation–CAAE Number 24911213.5.0000.5149). All volunteers received verbal information and signed a written informed consent prior to participating in this study.

## 3 RESULTS AND DISCUSSION

### 3.1 Yeast identification

The mean values of microbial load detected on SDA, for the three samples collected from workers (0, 30, and 180 days) and from waste (0 and 180 days), varied from 2.23 log·CFU/ml (day 0: workers' hands of the first shift work) to 0 (0 day: dental waste; 30th day: workers' nasal mucosa of the second shift work; day 180th: workers' hands and coats of the first shift work, worker's nasal mucosa, and coats of the second shift work). Table 1 exhibits the results of automated (Vitek^®^), conventional (slide culture and microscopy), and genotypic identification, and Table 2 displays the data obtained by the genetic approach. Findings generated by the three methods employed showed an agreement of 33.3%. Comparing data obtained from the employed methods with each other, automated to conventional methods, automated to genetic approaches, and conventional to genetic techniques, the agreement was of 50.0%, 38.9%, and 44.4%, respectively. In this study, there were three samples (#9, 13, and 18) that could not be identified by automated method (Vitek^®^). These three mentioned isolates were genetically identified as *Ogataea polymorpha*,* Rhodotorula mucilaginosa*, and *Candida albicans*, respectively. Other two samples (#5 and 11) could be identified by the automated Vitek^®^ system only at genus level. Sample #5 was identified as *Candida guilliermondii* by sequencing and sample #11 could not be identified at all. A recent study (Santos et al., 2016) compared DNA analysis, one automated method (API^®^/ID32), and fatty acid methyl esters profile as methods for yeast identification. Despite the differences between the studies design, the authors also reported low agreement among methods, when investigating samples recovered from environmental sources. When they compared the three methods, it was observed an agreement of only 16.6% and of 41.1% when API^®^/ID32 system and DNA analysis were compared, a result similar to the one obtained in the present study. The mentioned results disagreed with those published by Monteiro et al. (2016). The authors investigated 15 yeast samples recovered from blood culture and compared the identification obtained by Vitek^®^ and genotypic identification and found 100% of specificity. Their study design is somehow distinct from ours, where samples came from other sources as health care environment. In this study, DNA sequencing of four yeast strains (#6, 8, 11, and 17) exhibited low‐quality chromatograms (Phred values less than 20). As the intersimple sequence repeat (ISSR) region has been properly used to detect the intraspecific genetic differences (between fungi of same species), it could be used to show clonal population based on the bands pattern after electrophoresis (yeast 6 and 8 could be *Candida haemulonii*, # 11 could be *C. guilliermondii*, and # 17 could be *C. haemulonii*). The result of ISSR polymorphism is presented in the Supporting Information. The prevailing species identified by automated and conventional methodology was *C. haemulonii* (55.6%). According to Hou et al. (2016), *C. haemulonii* complex is an emerging group, which has been described as a cause of human infection including catheter‐related candidemia. In this study, the three used methods confirmed the identification of the samples #2 and 16 as *C. haemulonii*. The sample #12 was also identified as *C. haemulonii* by DNA and conventional methodology. The samples #2 and 16 were recovered from different sites of the same employee, the first one from the nasal mucosa and the second one from the professional clothing (belt line). This finding reinforces the importance of hand washing techniques to prevent health care‐associated infections. Genotypic technique is considered the gold standard methodology by Monteiro et al. (2016). This method demonstrated that *C. guilliermondii* was the prevailing species (4 samples) followed by *C. haemulonii* (3 samples), *Candida parapsilosis* and *C. albicans* (2 samples each), and *Cryptococcus victoriae*,* O. polymorpha*, and *R. mucilaginosa* (1 sample each). All of these species were previously recovered from dental solid waste by Vieira et al. (2010) except for *C. victoriae* and *O. polymorpha*. Górny and Dutkiewicz (2002) reported that yeast are frequently isolated from aerosols in indoor environments including health care settings. According to the authors, yeast could represent up to 52% of the fungi recovered from some investigated dwelling. Nunes, Bizerra, Ferreira, and Colombo (2013) reported that invasive infections caused by *Rhodotorula* species are mostly associated with underlying immunosuppression or cancer and the use of central venous catheters or other implantable medical devices. The authors also reported that the most frequent infection caused by *Rhodotorula* species is fungemia, followed by eye infections, peritonitis, and meningitis. Pitkäranta et al. (2008) reported that *C. victoriae* was among the dominant species recovered from indoor environments. The authors also compared culturing and DNA sequencing techniques to identify fungal species and suggested that cloning, cultivation, and quantitative PCR methods complemented each other, generating a more comprehensive picture of fungal content. The literature attested that the methylotrophic yeast *O. polymorpha* is one of the most important industrially applied yeast and could be isolated among others from soil, spoiled orange juice, leaf surfaces, exudates of plants, and insect guts (Suh & Zhou, 2010; Viigand, Visnapuu, Mardo, Aasamets, & Alamäe, 2016). The recovery of yeast species from waste workers' hands, nasal mucosa, and professional coats reinforces the need of frequent training programs inside health care institutions.

**Table 1:** Identification of 18 yeast isolates recovered from dental solid waste and waste‐handling workers' nasal mucosa, hands, and professional clothing, using automated, conventional microbiological, and genetic methods

ID	Sample^a^/employee^b^/workshift^c^	Site	Identification		
			Conventional	Automated^d^	Genetic
01	00/02/2s	C	*Candida parapsilosis*	*C*. *parapsilosis*	*C*. *parapsilosis*
02	30/06/1s	RNM	*Candida haemulonii*	*C*. *haemulonii*	*C*. *haemulonii*
03	00/04/2s	H	*C. parapsilosis*	*C. parapsilosis*	*C. parapsilosis*
04	30/06/2s	C	*Candida albicans*	*C. parapsilosis*	*Cryptococcus victoriae*
05	30/01/2s	C	*C. haemulonii*	*Candida famata*/*Candida guilliermondii* d	*C. guilliermondii*
06	30/06/1s	LNM	*C. haemulonii*	*C. haemulonii*	N.I.
07	30/06/2s	H	*C. guilliermondii*	*C. guilliermondii*	*C. guilliermondii*
08	00/04/1s	H	*C. haemulonii*	*C. guilliermondii*	N.I.
09	00/05/1s	H	*C. haemulonii*	N.I.	*Ogataea polymorpha*
10	30/04/2s	H	*C. haemulonii*	*C. guilliermondii*	*C. guilliermondii*
11	30/01/2s	H	*C. haemulonii*	*C. famata*/*C. guilliermondii* d	N.I.
12	30/06/1s	H	*C. haemulonii*	*C. guilliermondii*	*C. haemulonii*
13	00/06/2s	LNM	*Rhodotorula* sp.	N.I.	*Rhodotorula mucilaginosa*
14	—	W	*C. albicans*	*C. albicans*	*C. albicans*
15	30/01/1s	H	*Candida famata*	*C. famata*	*C. guilliermondii*
16	30/06/1s	C	*C. haemulonii*	*C. haemulonii*	*C. haemulonii*
17	30/05/2s	H	*C. haemulonii*	*C. haemulonii*	N.I.
18	180/06/2s	H	*Candida glabrata*	N.I.	*C. albicans*

ID, yeast isolate; C, coat; RNM, right nasal mucosa; H, hands; LNM, left nasal mucosa; W, potentially infectious waste; N.I., nonidentified.

a 00, first sample; 30, 30 days after the first sampling; 180, 6 months after the first sampling.

b 01 to 06, employee.

c 1s, first shift (morning); 2s, second shift (afternoon).

Vitek^®^(bioMérieux).

d Samples #5 and 11 were identified thrice as *C. famata*/*C. guilliermondii* (50% each).

**Table 2:** Data regarding genetic identification of 14 yeast isolates recovered from dental solid waste and waste‐handling workers' nasal mucosa, hands, and professional clothing

ID	Species	DNA sequencing			
		Bit score	E‐value	Identity	GenBank accession number
01	*Candida parapsilosis*	761	0.0	99%	KU739407.1
02	*Candida haemulonii*	499	1e‐137	98%	KU896954.1
03	*C. parapsilosis*	848	0.0	98%	KC966727.1
04	*Cryptococcus victoriae*	719	0.0	99%	KX349459.1
05	*Candida guilliermondii*	937	0.0	99%	KY104257.1
07	*C. guilliermondii*	968	0.0	99%	KY104257.1
09	*Ogataea polymorpha*	907	0.0	99%	KP675179.1
10	*C. guilliermondii*	948	0.0	99%	KY104257.1
12	*C. haemulonii*	475	2e‐130	100%	KU896954.1
13	*Rhodotorula mucilaginosa*	891	0.0	99%	KX866274.1
14	*Candida albicans*	739	0.0	98%	KY101906.1
15	*C. guilliermondii*	909	0.0	99%	KY104257.1
16	*C. haemulonii*	472	3e‐129	99%	KU896954.1
18	*C. albicans*	870	0.0	99%	KY101906.1

ID, yeast isolate.

### 3.2 Antifungal drug susceptibility

The minimal inhibitory concentrations (MICs) of tested antifungal drugs are depicted in Table 3. *Candida albicans* recovered from waste (#14) was nonsusceptible to voriconazole and fluconazole, *C. parapsilosis* (#1 and 3) and *C. guilliermondii* (#5) were susceptible to all drugs, and *C. haemulonii* (#6) was nonsusceptible to caspofungin and voriconazole. Orasch et al. (2014) reported that four *Candida* species (*C. albicans*,* C. glabrata*,* C. tropicalis*, and *C. parapsilosis*) represented more than 90% of all candidemia isolates in Switzerland, *C. albicans* remaining the predominant species. The authors attested that in vitro resistance to fluconazole, voriconazole, and caspofungin was rare among *C. albicans*, but an increase in nonsusceptibility rates was detected. In this study, MIC results for the *C. albicans* isolate recovered from waste proved a nonsusceptible profile to voriconazole (MIC > 64 μg/ml) and fluconazole (MIC > 16 μg/ml). Two samples identified as *C. haemulonii* (#2 and #6) recovered from the nasal mucosa of the same waste worker were nonsusceptible to caspofungin (MIC > 8 μg/ml). Additionally, the samples #2 and 8 were also considered as nonsusceptible to amphotericin B. Almeida et al. (2012) published the first Brazilian report involving a clinical isolate of *C. haemulonii* nonsusceptible to this antifungal drug. Studies attested that antifungal resistance is becoming an emerging problem and secondary resistance to amphotericin B has been described for several yeast species, including *C. haemulonii* (Alastruey‐Izquierdo, Melhem, Bonfietti, & Rodriguez‐Tudela, 2015; Ramos et al., 2015). Following the parameters established by CLSI M27‐A3 (Santos et al., 2014), samples #5 and 8 (identified as *C. guilliermondii* by genotypic and automated methods, respectively) and sample #17 (identified as *C. haemulonii* by conventional and automated methods) were susceptible to caspofungin (MIC ≤ 1). Sample #9 recovered from one waste worker and identified by internal transcribed spacer (ITS) DNA sequencing as *O. polymorpha* was susceptible to all five antifungals tested, according to the previous values informed by CLSI (CLSI, 2008). The results for *R. mucilaginosa* (#13), recovered from nasal mucosa of one waste worker, exhibited caspofugin MIC > 8 μg/ml. According to the CLSI, caspofungin MIC values of ≥8 μg/ml indicate resistance. Nunes et al. (2013) evaluated antifungal susceptibility profile of clinical and environmental isolates of *Rhodotorulla* spp. collected from 14 different Brazilian hospitals and found results similar to those presented herein. The value of fluconazole MIC for the sample #13 (≥64 μg/ml) was the same reported by these authors and considered by them “very high.” Antimicrobial susceptibility profile of the sample identified as *C. victoriae* by sequencing (sample #4) could not be determined by CLSI protocols despite two attempts (the yeast strain did not grow in RPMI).

**Table 3:** Susceptibility profile of 16 yeast isolates recovered from dental solid waste and waste‐handling workers' nasal mucosa, hands, and professional clothing

ID	Antifungals (μg/ml)									
	FLC		VOR		5‐FLU		CAS		AMB	
01	1	S	0.5	S	<0.125	S	1	S	1	S
02	8		4		<0.125	S	>8	NS	2	NS
03	1	S	0.5	S	<0.125	S	1	S	0.5	S
05	2	S	0.5	S	<0.125	S	2	S	0.5	S
06	8	S	4	NS	<0.125	S	>8	NS	1	S
07	32	S	8	S	<0.125	S	1	S	1	S
08	32	S	16	S	<0.125	S	0.25	S	2	NS
09	2	S	0.25	S	<0.125	S	0.125	S	0.25	S
10	0.5	S	0.5	S	<0.125	S	1	S	0.5	S
11	1	S	0.25	S	<0.125	S	1	S	0.5	S
12	4	S	2	S	<0.125	S	1	S	1	S
13	>64	NS	16	S	<0.125	S	>8	NS	0.5	S
14	>64	NS	>16	NS	<0.125	S	0.25	S	0.5	S
15	1	S	0.25	S	<0.125	S	0.5	S	0.5	S
16	8	S	2	S	<0.125	S	0.25	S	1	S
17	8	S	4	S	<0.125	S	2	S	0.5	S

ID, yeast isolate; FLC, fluconazole; VOR, voriconazole; 5‐FLU, 5‐flucytosine; CAS, caspofungin; AMB, amphotericin B; S, susceptible; NS, nonsusceptible.

### 3.3 Genetic investigation

The sequencing of ITS1‐5.8S‐ITS2 rDNA region was suitable to precisely identify 14 isolates to species level. High‐quality chromatogram regions that were chosen for sequencing analysis displayed values among 20–30 as judged by Phred software (http://www.phrap.com/phred/). These scores represent an accuracy of the base calling by sequencer reading of 99% and 99.9% (error probability of 1 in 100 and 1 in 1,000), respectively. DNA sequence chromatogram files judged by Phred <20 were not considered for molecular identification. The sequences submitted to online Blast tool (https://blast.ncbi.nlm.nih.gov/Blast.cgi) against GenBank database returned matched sequences with lengths between 268 and 534 bp, bit score greater than or equal to 472, E‐value less than or equal to 3e‐129, and identity greater than or equal to 98%, as shown in Table 2.

### 3.4 Genetic variability

**Figure 1:**
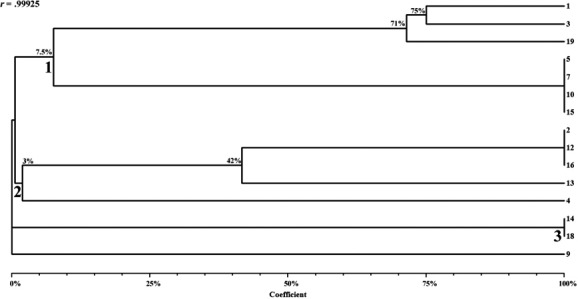
Dendrogram of the 14 yeast recovered from dental solid waste and waste handling workers' nasal mucosa, hands, and professional clothing. Yeast isolates 1–5, 7, 9, 10, 12–14, 15, 16, and 18 (according to Table 1), and 19: *Candida parapsilosis* ATCC 22019

PCR with (GTG) 5 oligo (Data S1) performed to investigate ISSR polymorphisms generated bright and defined DNA bands patterns in their majority, as well as allowed to design a dendrogram (Figure 1), displaying genetic distances. All bands were less than 3,000 bp. The dendrogram exhibited the presence of three major clusters (1, 2, and 3) and a *simplicifolious* chunck, composed by *O. polymorpha* (#9) isolate, based on similarity matrix and UPGMA grouping method, with correlation matrix value equal to 0.99925 (Figure 1). All employees, sites of sampling, and genetic relatedness of yeast strains recovered from waste and waste workers are depicted on Table 4. The cluster 1 was composed by *C. guilliermondii* (#5, 7, 10, and 15) isolates that displayed identical bands patterns and *C. parapsilosis* (#1, 3, and ATCC 22019) isolates that displayed intraspecific polymorphisms ranging from 71% to 75% similarity. The two branches of cluster 1 displayed low similarity between each other (about 7.5%). *Candida parapsilosis* isolates were recovered on the same day from different sites and employees working in the same shift. On the other hand, *C. guilliermondii* strains were isolated on the second sampling day from diverse sites and different employees working in opposite shifts. The cluster 2 was composed by *C. haemulonii* isolates (#2, 12, and 16), *R. mucilaginosa* (#13), and *C. victoriae* (#4). It was observed that *C. haemulonii* displayed identical band patterns, while they have exhibited 42% and 3% similarities with *R. mucilaginosa* and *C. victoriae* isolates, respectively. The three isolates of *C. haemulonii* were recovered from different sites of the same waste worker on the second sampling day. *Rhodotorula mucilaginosa* and *C. victoriae* isolates were recovered from the same afternoon employee on the first and second sampling days, respectively. According to Wirth and Goldani (2012), *Rhodotorula* is a common environmental yeast found in air, soil, lakes, ocean water, milk, and fruit juice. Despite previously considered nonpathogenic, *Rhodotorula* species have emerged as opportunistic pathogens with the ability to colonize and infect susceptible hosts. The isolation of one *Rhodotorula* strain from the nasal mucosa of one waste‐handling worker could be a matter of concern considering the possibility of cross‐contamination. In cluster 3, *C. albicans* (#14 and 18) isolates were grouped with 100% similarity; they have shown complete dissimilarity with yeast grouped in clusters 1 and 2. It is important to highlight this result once *C. albicans* #14 and 18 were both recovered on the last sampling (day 180), one from dental waste and the other from the hand of one waste worker. This finding suggests that the employee had been contaminated during his work activities and that waste should be considered as an important reservoir of microorganisms inside a health care setting. In turn, *O. polymorpha* (#9) was also completely dissimilar compared to any other isolated yeast result of ISSR (Figure 1). It was possible to observe that some yeast belong to the same clone and are circulating among waste workers. It was also confirmed that some strains were recovered from hands, nasal mucosa, professional clothing, and also inside dental waste. These findings prove that yeast isolates could be carried by waste workers that would be a source of infection especially for immunocompromised patients.

**Table 4:** Sampling and genetic relatedness of yeast strains recovered from waste and waste workers

Sample	Waste workers												
	Waste	1		2		3		4		5		6	
		FS	SS	FS	SS	FS	SS	FS	SS	FS	SS	FS	SS
0 Day	−	−	−	−	C^d^	−	−	H	H^d^	H	−	−	LNM
30th Day		H^a^	C^a^	−	−	−	−	−	H^a^	H	−	RNM^b^	C
												LNM	H^a^
												C^b^	
												H^b^	
180th Day	+^c^	−	−	−	−	−	−	−	−	−	−	−	H^c^

FS, first shift (morning); SS, second shift (evening); −, no yeast recovery; +, yeast recovery; C, professional coat; H, hands; LNM, lift nasal mucosa; RNM, right nasal mucosa.

a Samples had 100% similarity and belonged to the first cluster of dendrogram.

b Samples had 100% similarity and belonged to the second cluster of dendrogram.

c Samples had 100% similarity and belonged to the third cluster of dendrogram.

d Samples had 75% similarity and belonged to the first cluster of dendrogram.

## CONFLICT OF INTEREST

None declared.
