# Supplementary material for: Investigating cross‐contamination by yeast strains from dental solid waste to waste‐handling workers by DNA sequencing
Source: Microbiologyopen. 2017 Dec 26;7(2):e00554. doi: 10.1002/mbo3.554 (PMC5911987; doi:10.1002/mbo3.554)
Supplement: Supplementary file 1 [file MBO3-7-na-s001.docx]

**Supporting Information**: Polymerase Chain Reaction exhibiting ISSR polymorphisms of 14 yeasts recovered from dental solid waste and waste handling workers’ nasal mucosa, hands and professional clothing.


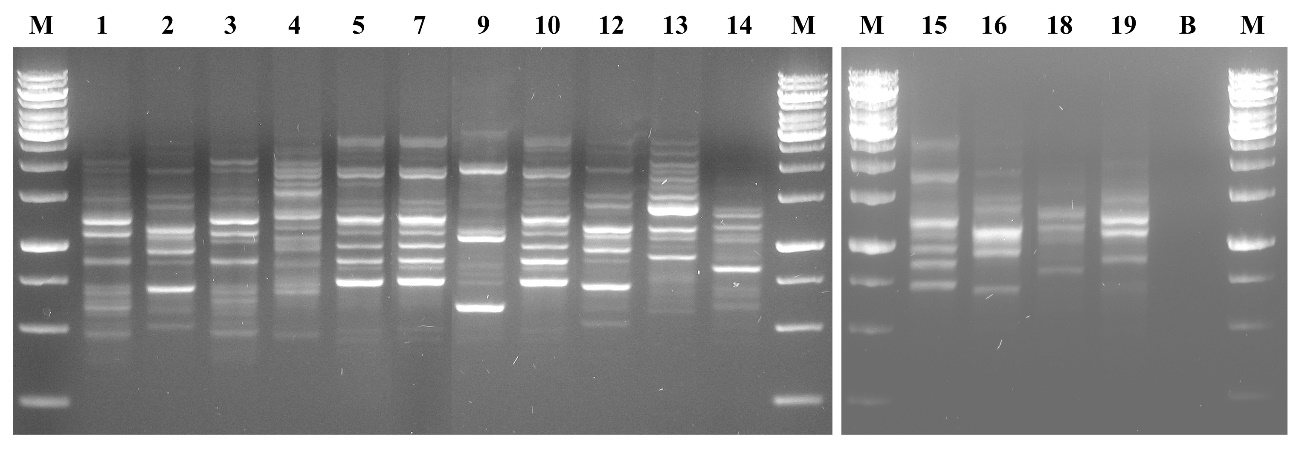


M: DNA ladder; 1-5, 7, 9, 10, 12-14, 15, 16 and 18: yeast isolates (according to Table 01; 19: *Candida parapsilosis* ATCC 22019; B: negative control (mix without DNA template).
